# The Relations of Dental Science to Medicine

**Published:** 1876-02

**Authors:** 


### THE RELATIONS OF DENTAL SCIENCE TO MEDICINE.

ADDRESS BY PROF. HAMMOND.

Mr. President and gentlemen of the American Academy of Dental Surgery:—I am very sure that I do not presume too much when I venture to say to you that the cordial sympathy of the medical profession is with you in the effort in which you are engaged. Medical science is a liberal science; at any rate, it professes to be such; it does not hesitate to embrace in its fold all those who are interested in or devoted to the alleviation of the sufferings of mankind, and I see no reason why those who practice the profession of dentistry should not occupy one of the highest of all professions; because certainly if we look to the ills which will follow derangements of the teeth and to the good which is constantly being accomplished in the alleviation of these ills, we are bound to accord to dentistry a very high position with those engaged in similar labors, At one time there was no difference between the dentist and the surgeon, they occupied a common ground—very much undoubtedly to the disadvantage of their patients; because if anything tends to the advancement of any separate department of medical science, it is the separation of that department from the mass by those who take a special interest in it. Prior to the separation of dentistry from surgery proper, not much progress was made in any one scientific department. I have taken the liberty of bringing here a volume now some three hundred years old, written by Ambroise Pare; and it is only necessary to read from this volume to see how they performed dental operations in his day.

For instance, in a chapter on the toothache, he gives as a special remedy the taking of oil of vitriol into the tooth, and if that does not succeed, the burning of it with the hot iron. [The doctor then proceeded to read a number of extracts from the volume, showing the state of dental knowledge three hundred years ago. From the standpoint of modern science, the way of the old surgeon's directions seemed ludicrous indeed; while his quaint remarks produced much laughter.]

I think, gentlemen, that dentistry would be much better if dentists were qualified surgeons, if they had not separated themselves from surgery proper. We may differentiate too much. Have we not, in simply striving to enlarge the bounds of science, to some extent cut loose from the pathway through it? As to whether it is wise to do so or not, we are not the best judges. That there is no actual differentiation between medicine and dentistryis very evident; but practically it may amount to something of the same kind. So the question arises in my mind, whether it is wise to have dental colleges; whether it will not be better to have chairs of dentistry in medical colleges, where students who followed the lectures, would not only become proficient in dentistry, but obtain a full knowledge of medicine, without which it strikes me that no man can become an accomplished dentist.

That brings me to the consideration of the relations which naturally exists between the two sciences. I have no doubt that my friend Dr. Carroll has gone very thoroughly over the ground which, to some extent, it is necessary for me to go. We have one common ground between us—the fifth pair of nerves. All surgeons work upon it, more or less. All dentists work upon it entirely. Is the dentist who is simply a dentist (who knows the teeth and nothing, else, and has not studied the whole intricate ramifications which science will lead him to, in anatomy, physiology, pathology, therapeutics) is he competent to point out to his patients what they are to do? You will agree with me that he is not, that it is necessary for him to have some general knowledge of the functions of the nervous system and the functions of the body as a whole, of the therapeutical value of drugs and of general pathology.

Here we have the fifth pair of nerves, with its power of motion and power of sensation. We may have various diseases manifested in different parts of the body from the irritation set up at one point. It is part of the special branch of medical science, in which I happen to be engaged, to meet with patients who are suffering from nervous affections which are clearly to be ascribed to affections of the teeth. Only a few days ago I had a young lady patient who was suffering from paralysis of the fifth pair of nerves. Her physician said it was internal strabismus. She was too young to have over worked her nervous system to any great extent and there were no other symptoms. She was suffering from one of her molar teeth, which was decayed, It was removed and the paralysis ceased in twenty-four hours. I saw only a short time ago a very interesting case of neuralgia, which was entirely cured by rearranging the teeth. You all agree with me that the face is specially liable to be excited by irritation of the fifth pair. It would not be worth while for me to go fully into the various diseases which may be excited in different parts of the body by affections of the teeth.

The relation of medicine to dental science are not only very manifest as regards the prominent diseases which we have to treat, but likewise to the common contributions which have been made to the common science of medicine. I suppose, considering the little field that the dentists occupy, that they have contributed more to anatomy, histology and therapeutics than any other class of medical men. When we come to the histology of the teeth, we leave it entirely to the dentists. Then they gave us that great boon of anæsthesia. Dentists have been very large contributors to the science relating to diseases of the jaw, palate, teeth and tongue.

I said in my opening remarks that I was inclined to think that the dentists have been probably indiscreet in separating themselves too much from surgery proper. They could study every branch of dentistry just as well and still be doctors of medicine. At the present day I think that no such differentiation should be made. I think this separation a bad move and that it is not carried in Europe to the same extent as it is in America. I think a surgeon who prefers to practice one branch of surgery is just as much a surgeon as he who practices general surgery.

You have asked me here to give my views relative to the relations which exist between dental science and medical science. I am for making them closer than they ever were.

